# Transforming care by integrating maternity and psychological support: a mixed-methods evaluation of a Maternal Mental Health Service

**DOI:** 10.1186/s12884-025-08096-9

**Published:** 2025-10-17

**Authors:** Jasmine Reed, Hadiss Khossravi, Sanne van Rhijn

**Affiliations:** 1 Maternity Trauma & Loss Care Service, West London NHS Trust, London, UK; 2 Children and Young Persons’ Community Eating Disorder Service, Springfield University Hospital, London, UK; 3 Perinatal Mental Health Service, West London NHS Trust, London, UK; 4 Department of Brain Sciences, Imperial College, London, UK

**Keywords:** Trauma-informed, Pregnancy, Perinatal, Childbirth, Treatment, Evidence-based, Psychology

## Abstract

**Background:**

Maternal mental health difficulties, associated with perinatal loss, a traumatic birth or fear of childbirth, are common and yet often left untreated. Maternal Mental Health Services were set up as part of the National Health Service (NHS) Long Term Plan to address this gap and provide psychological treatment and specialist midwifery care to women and birthing people experiencing maternal mental health difficulties using trauma-informed frameworks. This study aimed to evaluate the establishment and initial treatment outcomes of the Maternity Trauma & Loss Care Service (MTLC), a Maternal Mental Health Service, for women and birthing people affected by perinatal trauma, loss, and tokophobia.

**Method:**

The sample includes all women and birthing people (*N* = 254) referred to the Maternity Trauma & Loss Care Service over the 12-month period from 1st April 2022 to 31st March 2023. Data were analysed using a mixed‐methods cross‐sectional design.

**Results:**

Of the 72 clients with completed outcome measures, a reduction in the proportion of clients reporting clinically significant levels of psychological distress between the start and end of treatment from 77 to 29% was observed. The proportion of clients reporting clinically relevant symptoms of post-traumatic stress was reduced from 80% pre-treatment to 15% post-treatment. The 17 clients who provided feedback, overall reported that, as a result of accessing the service, they felt supported, listened to and advocated for whilst they received maternity care.

**Conclusion:**

This study provides useful insights about Maternal Mental Health Services, that they fill a clear gap for women and birthing people with mental health difficulties related to pregnancy, birth and perinatal loss. It highlights areas for future research and barriers for implementing these services.

**Supplementary Information:**

The online version contains supplementary material available at 10.1186/s12884-025-08096-9.

## Introduction

Pregnancy and childbirth, for many women and birthing people, is a positive, life-changing experience. However, although maternal mental health problems are common, they are often left untreated and associated with adverse obstetric and neonatal outcomes [1]. Perinatal mental illness carries a cost to society of £8.1 billion each year in the United Kingdom and approximately three-quarters of this cost relates to adverse impacts on the child, including costs to health and social care [2]. In a follow-up report, the net benefit of an integrated service provision to address the unmet needs of women with common mental health problems in the UK was an estimated £55 million each year [3].

Several contributing factors have been identified in the development of maternal mental difficulties. Firstly, 20–40% of women and birthing people consider labour and birth a traumatic experience [4, 5], with around 4% developing Post-Traumatic Stress Disorder (PTSD) [6]. This in turn has been associated with an increased risk of postnatal depression and impaired bonding with the infant [7, 8]. Identification and treatment of PTSD is therefore imperative to improve maternal health outcomes and potentially prevent the longer-term consequences for both parent and child. In this article we will be referring to perinatal trauma as trauma that can occur at any stage in the maternity journey from conception to the postnatal period, including a traumatic birth [9].

A prominent anxiety disorder related to childbirth is tokophobia, defined as a severe fear of pregnancy or childbirth, with a prevalence of over 20% in Western Countries [10–12]. Tokophobia can precede conception and lead to avoidance of pregnancies or arise following a traumatic birthing experience [13].

Risk factors for a traumatic birth include fear of giving birth [14], actions of health care providers during labour and on the postnatal ward, and historical abuse or trauma [15] Though, subjectively perceived traumatic births have often been viewed as ‘routine’ by clinicians [16], feeling ignored, unsupported or abandoned by healthcare professionals have been identified as moments of extreme distress implicated in the development of PTSD symptoms [17]. Systemic racism has also been frequently highlighted as a risk factor for traumatic births; women and birthing people from ethnic minority backgrounds are more likely to experience trauma due to particularly poor care, as well as racism and discrimination from services [18]. Subsequently, those from an ethnic minority are more likely to report clinically significant PTSD symptoms after childbirth [19].

Another contributing risk factor for maternal mental health difficulties is experiences of perinatal loss [15, 20] which affects millions of individuals worldwide each year [21]. Perinatal loss includes miscarriage (foetal death prior to 24-weeks’ gestation), stillbirth (foetal death after 24 weeks’ gestation), or neonatal death (death between birth and 28 days after birth). Perinatal grief has repeatedly been identified as a predictor of PTSD [22, 23]. As the majority of women and birthing people conceive again following a pregnancy loss, PTSD symptoms can occur during subsequent pregnancies [24] and may contribute to difficulties bonding with the unborn baby [20, 25]. Women and birthing people with a history of pregnancy loss also experience higher levels of anxiety during subsequent pregnancies compared to those without prior loss [26].

Awareness of, and sensitivity to, previously experienced trauma is being increasingly recognised as crucial for obstetric care [27]. Trauma Informed Care (TIC) describes considering and delivering care which is sensitive to the needs of clients and staff who may have experienced trauma [28]. The aim of TIC is to reduce re-traumatisation and relies on principles of safety, choice, collaboration, empowerment and trustworthiness [29]. TIC has been identified to both reduce adverse outcomes in maternity settings [30] and improve bonding with baby [31].

Whilst TIC has increasingly been introduced in obstetric care, National Health Service (NHS) England and NHS Improvement consultations identified a considerable group of women and birthing people requiring specialist mental health support related to their maternity experience, which was beyond existing service provisions. It was recognised that in many cases the current provision of care through Community Perinatal Mental Health Services and NHS Talking Therapy services did not include provision of care for complex perinatal trauma, perinatal loss and tokophobia [32]. As a result, Maternal Mental Health Services (MMHS) were set up in the UK as part of the NHS Long Term Plan [33]. MMHS’ are specialist perinatal mental health services, guided by trauma informed frameworks, delivering integrated maternity, reproductive health and evidence-based psychological therapies.

Early implementer and fast follower sites were initially set up across England. Transformation funding of £22.6 million was available for Early Implementer and Fast Follower sites piloting the MMHS models in 2020/21 and 2021/22. The ambition of the plan was to have at least 66,000 women with moderate to severe perinatal mental health difficulties access MMHS specialist care by 2023/24 [34].

The current study aims to provide preliminary data about a pilot MMHS (MTLC) in its first year, objectives are to: (i) understand if the MLTC service is associated with women and birthing people’s improved psychological outcomes following perinatal trauma, loss and tokophobia as measured by the CORE-10 and the PCL-5; and to (ii) understand if women and birthing people were satisfied with the service they received, focusing on their experiences of support, care and perceptions of its effectiveness. This research shares initial outcomes of evidence-based psychological treatment and psychologically informed midwifery support for women and birthing people who experienced a traumatic birth, perinatal loss, pregnancy after loss or tokophobia.

## Methods

### Design & procedure

The study used a mixed methods design. This service evaluation was approved by the West London NHS audit committee. We followed the UK Health Research Authority decision tool to determine that research ethics approval was not required, as the project entailed a retrospective, anonymised analysis of routinely collected service data. Demographic data was obtained from online electronic client Rio records.

### Setting

**Fig. 1:**
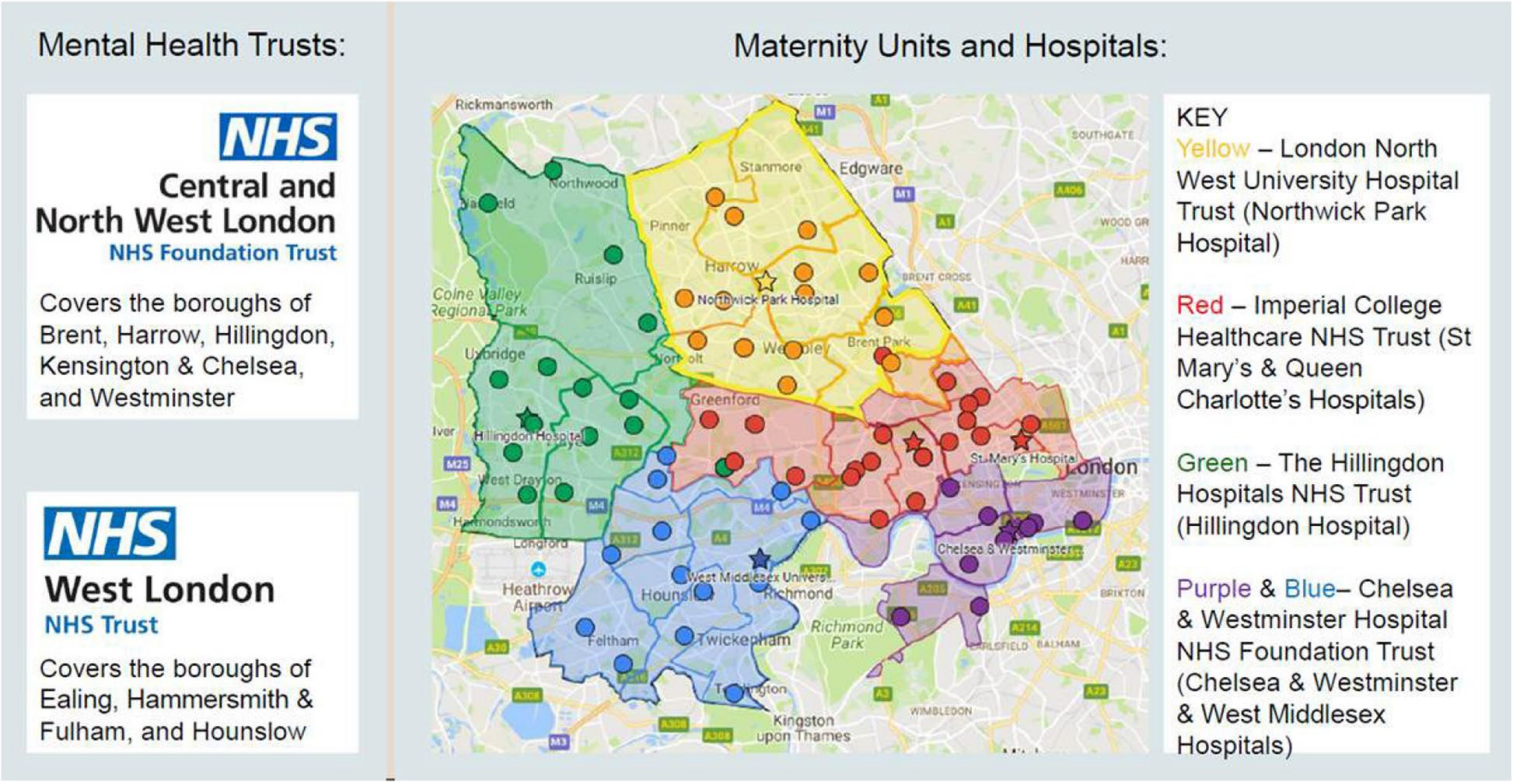
Map of MTLC coverage area

The North-West London (NWL) Maternity Trauma and Loss Care (MTLC) service is a MMHS that covers the eight NWL boroughs, as seen in Fig. 1, and comprises of two ‘sub teams’; the West London and the Central and Northwest London MTLC Service. This study sample will focus on the West London MTLC service, which covers the boroughs of Ealing, Hounslow and Hammersmith & Fulham, hereby referred to as ‘MTLC’. West-London has an ethnically diverse population; Ealing’s population is nearly 30% Asian, Asian British or Asian Welsh and nearly 11% is Black, Black British, Black Welsh, Carribean or African. Similarly, Hounslow’s population is almost 37% Asian, Asian British or Asian Welsh, and 7.2% identifies as Black, Black British, Black Welsh, Carribean or African. A larger proportion of the population identify as White in Hammersmith & Fulham (63.2%), with a smaller representation of Asian (10.5%) and Black British, Black Carribean or African (12.3%) [35]

MTLC has an integrated team of specialist perinatal mental health midwives and psychological practitioners. The service accepts self-referrals and referrals from any other healthcare professionals, such as maternity, mental health teams and GPs.

The MTLC service is for individuals who have experienced a traumatic pregnancy or birth, perinatal loss (during pregnancy or within the first year of life), or have tokophobia (with or without previous experience of pregnancy). The eligibility criteria for referrals to MTLC include women and birthing people who are aged 18 or over and living within the West London geographical boundary. They must also either be currently pregnant or have been pregnant, given birth or had a perinatal loss within the last 12 months. If currently pregnant, clients need to be booked for their maternity care at a North-West London maternity hospital. Clients are seen in a variety of different clinic settings including maternity hospitals, children centres, GP surgeries and online via MS Teams.

### Sample

The sample includes all clients who were referred to MTLC over the 12-month period from 1 st April 2022 to 31 st March 2023. The service received 254 referrals. Of the 254 referrals MTLC received, 14 referrals were re-referrals within the year i.e. referrals for clients that had already been referred within April 2022 and March 2023. Thus, 240 people were referred to MTLC in total. Of the 190 accepted referrals, 5 individuals were referred twice, accepted and offered an assessment. Therefore 185 clients were referred to MTLC and offered an assessment. Of these, 72 clients had paired, completed outcome measures and were included in the analysis.

### Interventions

MTLC offers short-to-medium term interventions; up to 8 midwifery sessions and up to 24 psychological therapy sessions. MTLC aims to take an active role in preventing re-traumatisation and empowering women and birthing people throughout their maternity journeys.

One-to-one specialist midwifery care includes building trusting relationships with clients, offering evidence-based interventions to manage anxiety and fear, and help navigating the maternity system. The evidence-based support [36] also includes trauma-informed birth preparations where a collaborative approach aids the client and clinician to consider how to minimise the activation of previous trauma experiences, how to address labour-related challenges such as the client’s need for control and difficulties with disclosure. This is achieved through the MTLC midwives advocating for clients, as well as enabling clients and their birthing partners to advocate for themselves during pregnancy and childbirth. These factors are essential to create a positive birthing experience [37]. MTLC midwives also support clients in navigating complex maternity systems and advocating on their behalf during interactions with wider NHS services. Midwives working within MTLC are employed by the acute hospital trusts but hold honorary contracts with affiliated mental health trusts. Their roles are protected for exclusive delivery of MTLC care; thus, they do not undertake routine clinical duties within the acute maternity setting. This employment model facilitates the integration of psychologically informed midwifery practice within acute services.

The psychological interventions offered comprise NICE recommended and concordant interventions for perinatal trauma and loss. This includes Trauma-Focused Cognitive Behavioural Therapy (TFCBT), Eye Movement Desensitisation and Reprocessing (EMDR), Compassion Focused Therapy (CFT) and Acceptance and Commitment Therapy (ACT).

Trauma focused therapy is a specialised form of psychotherapy which helps individuals process and heal from traumatic experiences. The goal is to create a safe environment where clients can explore their feelings, understand the impact of trauma on their lives, and develop healthier coping mechanisms to help reduce symptoms such as anxiety, flashbacks, and emotional numbness. The benefits of trauma therapy are profound. It can enhance emotional resilience, improve relationships, and restore a sense of safety and control. Many individuals experience reduced PTSD symptoms, greater self-awareness, and an improved ability to handle stress [38].

All antenatal clients were offered an intervention of midwifery support, unless deemed not necessary, for example if they were receiving enhanced midwifery care from their maternity hospital or were more closely monitored by medical or obstetric teams. Where psychological needs were identified—either at initial assessment or during the antenatal period—clients were also offered psychological therapy alongside midwifery support. This integrated approach enabled coordinated care that addressed both the psychological and maternity-related needs of clients, promoting shared understanding and partnership between professionals and clients.

Guided by the NHS-endorsed TIC principles for perinatal mental health [39] —Compassion and Recognition, Communication and Collaboration, Consistency and Continuity, and Recognising Diversity and Facilitating Recovery, MTLC integrates these principles into all levels of its clinical delivery. MTLC places high value on transparent, two-way communication, enabling clients to make informed choices about their care.

The foundation of MTLC’s model is compassion that recognises the pervasive impact of trauma on the perinatal experience. Staff are trained to understand how trauma may shape a person’s engagement with care, even in the absence of disclosure. MTLC midwives offer one-to-one support, building therapeutic relationships through trust, emotional presence, and attuned listening.

Continuity of care is not routinely available in many maternity settings yet is a key protective factor against re-traumatisation. MTLC midwives, provide consistent, ongoing care from referral to postnatal discharge. This stability allows clients to avoid repeated disclosures of trauma and supports the development of trust over time.

The service actively engages with individuals whose needs are often underserved—those affected by perinatal loss, refugee and migrant communities, and people experiencing tokophobia. Culturally sensitive approaches and personalised care planning help address the varied ways in which trauma is experienced and expressed. Clinicians are encouraged to hold space for complex grief and identity formation during the perinatal period, especially for those with intersecting vulnerabilities such as past abuse, systemic racism, or socioeconomic disadvantage. This principle is also extended to the workforce, acknowledging that staff themselves may carry trauma histories, and offering reflective practice to mitigate vicarious trauma and compassion fatigue.

### Demographic information

Demographic data collected included: ethnicity, borough of residence, pregnancy status, booking hospital and referrer.

### Measurements

#### Clinical outcomes in routine evaluation (CORE-10)

The CORE-10 [40] is a 10-item patient-rated measure that considers both experienced symptoms of low mood, anxiety and functioning (ability to cope, availability of help) in the last week. While the validity in different ethnic groups has not been formally established, the measure is routinely used in the NHS for diverse populations. Responses are measured on a 5-point Likert scale: 0 (not at all) to 4 (most or all of the time). The score range is 0–40, with higher scores indicating higher distress. The CORE-10 was collected at assessment, before intervention, and at discharge from the MTLC service by the clinician carrying out the intervention (psychological therapist or midwife).

#### Post-traumatic stress disorder checklist for DSM-5 (PCL-5)

The PCL-5 [41] is a 20-item patient-rated measure that assesses the 20 DSM-5 symptoms of PTSD. The PCL-5 asks respondents how often they have been bothered by a symptom in the past month. Responses are measured on a 5-point Likert scale: 0 (not at all) to 4 (extremely). The score range is 0–80, with higher scores indicating higher distress. A PCL-5 score of 33 and above suggests a high likelihood of PTSD. The PCL-5 has a high demonstrated high internal reliability (α = 0.94) [42]. It was selected as an outcome measure due to its demonstrated validity in ethnically diverse groups [42]. The PCL-5 was collected at assessment, before intervention, and at discharge from the MTLC service by the clinician carrying out the intervention (psychological therapist or midwife).

#### Service user satisfaction survey

This survey was sent to all clients following treatment discharge. It is based on the NHS Friends and Family Test and was co-produced by experts by experience, who were part of mobilising the service. The survey asks clients to provide feedback on their experience of MTLC. Seven questions were quantitative in nature, with ratings determined on a five-point Likert-type scale by level of satisfaction ranging from unsatisfied to very satisfied, including a neutral option. Below these seven questions was the option to provide qualitative feedback on the topic the question asked about the service. For example, one question was ‘How much do you feel you were treated with kindness and respect by the staff in M-TLC?’ with the option to give a ranked response and an option to leave qualitative feedback. The survey further includes questions regarding treatment by staff, involvement in care and decisions, impacts of the support received from the service on emotional and mental wellbeing, and satisfaction with waiting times.

### Analysis

Deprivation was assessed using the index of multiple deprivation (IMD) based on the postcode of the client [43]. Areas were assigned to deciles, where decile 1 represents the most deprived 10% of areas in England, and decile 10 represents the least deprived. For analysis, IMD deciles were grouped into three bands: among the 20% most deprived areas (deciles 1–2), among the 20–40% deprived areas (deciles 3–4), moderate deprivation (deciles 5–6), among the 20–40% least deprived areas (deciles 7–8), among the 20% least deprived areas (deciles 9–10).

Quantitative data including CORE-10 and PCL-5 scores, were extracted from the service electronic database RiO. Quantitative data that could not be extracted from the electronic databases were manually recorded by staff. Where required, data were cross referenced with individual client records. Confidentiality was respected and upheld during both the analysis and dissemination. Descriptive statistics for the referrals received were analysed reviewing the description of the clients’ demographic information, such as reason for referral, interventions offered and client ethnicity. Paired sample T-Tests were performed using IBM SPSS Statistics, version OS for Mac [44]. Qualitative feedback was gathered through completion of the Service User Satisfaction Survey. Recurrent themes throughout the surveys were identified and summarised.

## Results

### Characteristics of the sample

MTLC received 254 referrals between April 2022 and March 2023. Of those, 190 referrals (75%) were accepted and 64 (25%) were not accepted. Of the 185 clients referred to MTLC and offered an assessment, primary referral reasons included: 92 (50%) due to perinatal trauma, 65 clients (35%) for perinatal loss, 26 (14%) for tokophobia and 2 for other reasons (1%).

Of clients offered an assessment with the service, 25% were White British, 22% were from other White backgrounds, 19% were Asian or Asian British and 9% of clients were Black or Black British (see Supplementary Table, Additional File 1). The acceptance rates were largely consistent between individuals from White British backgrounds and individuals from minority groups.

**Fig. 2:**
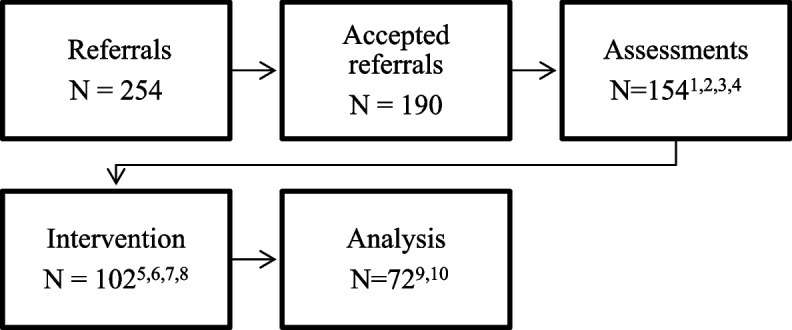
Flowchart demonstrating outcomes of referrals to analysis. ^1^ 21 clients did not opt in to the service. ^2^ 8 clients did not attend the assessment. ^3^ 2 declined the service. ^4^ 5 assessments not conducted due to other circumstances. ^5^ 39 were assessed and signposted/received an onward referral. ^6^ 6 were assessed and no longer met criteria for MTLC. ^7^ 3 Experienced a miscarriage following the assessment with the option to re-refer. ^8^ 4 disengaged following the assessment. ^9^ 28 clients disengaged with the service. ^10^ 2 clients lacked follow-up data

Figure 2 shows of the 190 accepted referrals, 31 (17%) did not opt in to the service, did not attend the assessment or declined the service and 5 assessments were not conducted due to other circumstances. Thus, 154 assessments occurred. After these assessments, 39 clients (21%) were signposted or referred on to another service. A small number were assessed and no longer met criteria or declined support from MTLC thus were discharged. A few disengaged following the assessment or experienced a miscarriage following the assessment and requested discharge or signposted to a more appropriate service. Of the 102 offered an intervention, 28 disengaged with the service and two clients lacked follow-up data. This meant 72 clients were included in our analysis.

Table 1 provides a summary of demographic and clinical characteristics of clients included in the analysis. Clients accessing interventions were evenly distributed across all grouped IMD ranks, with no clear overrepresentation from any level of deprivation. Three clients did not have recorded deprivation data available regarding their postcode. At referral, almost three quarters of clients (74%) who were assessed, offered an intervention and engaged in treatment were antenatal. A small number (6%) were postnatal, eight (11%) were referred for perinatal loss and were not pregnant and five (7%) were referred for perinatal trauma and not pregnant. At discharge, almost all clients (93%) were postnatal, four (6%) were not pregnant (post-loss) and one was pregnant. Adjustment disorder, the diagnostic category used for perinatal loss in the absence of PTSD, was the most common diagnostic category used (*n* = 33(46%)). Eighteen clients (25%) had a diagnosis of PTSD, and nine clients (13%) of other phobic anxiety (the code used for tokophobia). The remainder of clients received a diagnosis of other types of anxiety disorder.

**Table 1:** ICD-10 diagnosis, pregnancy status, index of multiple deprivation rank and ethnicities of analysis sample (*n* = 72)

Pregnancy Status	At referral	At discharge
Not pregnant (post-loss)	8 (11.1%)	4 (5.6%)
Not pregnant (perinatal trauma)	5 (6.9%)	0 (0%)
Pregnant	53 (73.6%)	1 (1.4%)
Postnatal	6 (8.3%)	67 (93.1%)

ICD-10 Diagnosis		
Adjustment disorder	33 (45.8%)	
Post Traumatic Stress Disorder	18 (25.0%)	
Other Phobic Anxiety Disorder	9 (12.5%)	
Unspecified Anxiety Disorder	5 (6.9%)	
Specific phobia	5 (6.9%)	
Generalised Anxiety Disorder	2 (2.8%)	

Index of Multiple Deprivation Rank [43]		
Among the 20% most deprived areas	8 (11.6%)	
Among the 20–40% most deprived areas	19 (27.5%)	
Moderate deprivation	17 (24.6%)	
Among the 20%−40% least deprived areas	20 (29.0%)	
Among the 20% least deprived areas	5 (7.2%)	

Ethnicity		
White—British	18 (25.0%)	
White—Other	15 (20.8%)	
Asian – Indian	7 (9.7%)	
Asian—Other	6 (8.3%)	
Black or Black – British	7 (9.7%)	
Black—Other	1 (1.4%)	
Mixed – Other	7 (9.7%)	
Other	5 (6.9%)	
Not Stated	6 (8.3%)	

### Interventions

Of the interventions offered, 50 (49%) were offered a midwifery intervention, 32 (31%) a psychology intervention and 18 (18%) were offered joint midwifery and psychology interventions. A small number attended groups.

### Quantitative results

#### Core-10

**Fig. 3:**
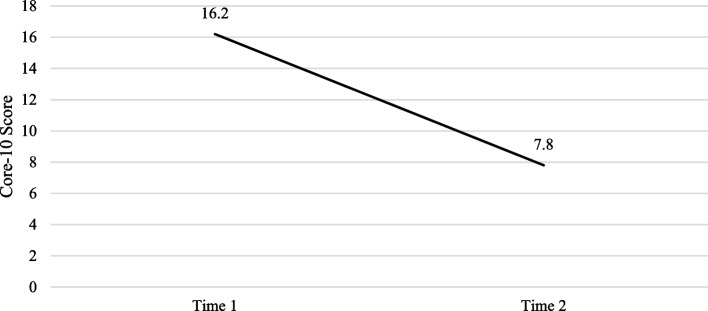
Core-10 scores. Average scores on the Core-10 at time 1 and time 2 for clients in treatment in the MTLC service April 2022- March 2023

Table 2 shows a total of 72 clients who completed the CORE-10 both prior to and after completing treatment. Before treatment (T1), 76.5% of clients in the service had clinically significant scores on the Core-10. Following treatment (T2), this decreased to 29.2%. Mean scores decreased from T1 to T2, and paired samples t-tests showed this to be significant *(t (71)* = *10.486, p* < *0.001)*. This indicated a significant reduction in symptoms of psychological distress, as seen in Fig. 3.

**Table 2:** Core-10 & PCL-5 scores

Core 10 (*n* = 72)			PCL (*n* = 20)		
T1 M (SD)	T2 M (SD)	p	T1 M (SD)	T2 M (SD)	p
16.19 (7.99)	7.78 (7.63)	<.001	42.50 (17.49)	18.00 (13.00)	<.001

#### PCL-5

**Fig. 4:**
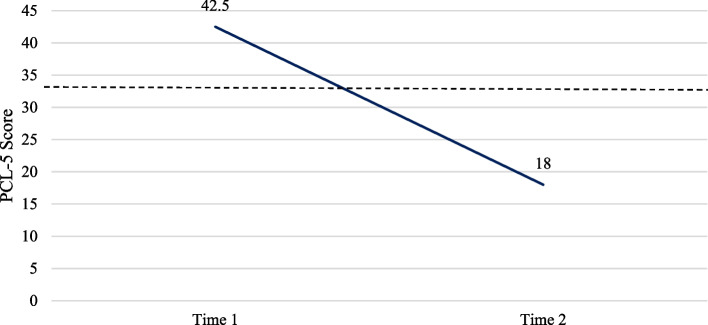
PCL-5 scores. Average scores on the PCL-5 at time 1 and time 2 for clients in treatment in the MTLC service April 2022- March 2023. The dotted line indicates the clinical cut off score of 33

Completion of the PCL-5 at assessment and during or after treatment was only deemed appropriate for clients presenting with PTSD symptoms. Table 2 shows 20 clients completed the PCL-5 both prior to and following treatment. At time 1, 80% of clients in the service had clinically significant scores on the PCL-5. Following treatment, this decreased to 15%. Mean scores decreased from T1 to T2, and paired samples t-tests showed this to be significant *(t (19)* = *32.699, p* < *0.001)*. This indicated a significant reduction in PTSD symptoms, as seen in Fig. 4.

#### Service user satisfaction survey

The service received 17 positive submissions, 0 neutral and 0 negative submissions of the Service User Satisfaction Survey.

All clients who completed the Service User Satisfaction Survey felt they were very involved in making decisions about their care and treatment whilst under the MTLC service. All responses indicated clients felt they were very much treated with kindness and respect by MTLC staff. Responses showed that 94% of clients were very satisfied with the waiting time from referral to assessment and 94% felt satisfied with the venue where they had their MTLC appointments. Responses also showed that 68% of clients were very satisfied with the information provided about MTLC before the first appointment. All clients who filled in the survey felt their questions about their care and treatment were answered very well by staff within the MTLC service. To note, 88% of clients who filled in the survey, felt that engaging with the MTLC service meant their emotional and mental wellbeing improved.

### Qualitative feedback

Key themes identified from the free text responses included: experiencing staff as supportive and attentive, feeling increased confidence and safety, feeling valued by midwives advocating for them in maternity spaces. Clients also expressed hope that the service will continue to expand. We were not able to do a structural analysis due to limitations in the material.

#### Staff are supportive and attentive

“I was given an open space to discuss how I felt and honour my baby, who was forgotten by everybody else and for that I am forever thankful.”

“I have been treated with kindness. I felt respected and valued.”

#### Feeling increased confidence and safety

“Seeing [my clinician] regularly helped me to build up my confidence and prepare for my baby which made me happy. I started to worry less and look forward to seeing my baby.”

#### Valuing midwives advocating for them in maternity spaces

“It made me feel more in control of my pregnancy and labour and more at peace/reassured once the birth came. The birth plan I developed with [my clinician’s] help made me feel more confident and safer during labour.”

“[My clinician’s] inside knowledge of how and when to get in touch with my doctors and how to navigate through pregnancy was invaluable”

“I credit [my clinician] completely with the positive birth experience I had – she was pivotal in making the arrangements that led to the wonderful memories I have of the birth of my daughter”

#### Hope that the service will continue to expand

“I would love to see the service expand. It would be great to see some occupational therapists in the team to support new mums with getting back into their day-to-day activities whilst developing a new role and identity with lots of new routines and daily activities.”

“I hope MLTC is able to continue to further assist women such as myself to have positive birth experiences”

## Discussion

This study aimed to analyse preliminary data about the MTLC Service, a MMHS, and share initial quantitative and qualitative findings. To our knowledge, an evaluation of a MMHS using TIC, has not been carried out to date.

Women and birthing people referred to the service were from a diverse range of ethnic backgrounds. It was found that the service’s acceptance rate for individuals from Black, Asian and Minority Ethnic populations is in keeping with the services overall acceptance rate. The acceptance rate further indicates that of the clients referred, Black, Asian and Minority Ethnic populations women were not less likely than their white peers to be offered an assessment or intervention. Offering an assessment and trauma-informed intervention to women and birthing people from diverse backgrounds, including from different areas of deprivation, and potentially intersecting vulnerabilities, is crucial for ensuring equitable access to MMHS and TIC with a view to improve maternal and infant health outcomes. Given that Black, Asian, and minority ethnic women experience higher rates of birth trauma, maternal mortality and adverse outcomes compared to white women, trauma-informed, culturally sensitive interventions can help mitigate these disparities by addressing specific needs and challenges faced by these communities [45].

Over half the clients referred (54%) were offered a trauma-informed intervention. Of those offered interventions, half received an intervention of midwifery support, just less than a third received psychological therapy and 18% received both psychological therapy and midwifery support. The psychological therapies offered, of EMDR and TF-CBT, were NICE guideline recommended, and the midwifery support was evidence based [34]. This demonstrates that MTLC is meeting the previously unmet maternal mental health needs, in particular of a trauma-informed care approach, of a significant part of the birthing population in the local area.

The findings demonstrate that majority of clients experienced a significant reduction in overall psychological distress; symptoms of PTSD, low mood and anxiety reduced following treatment. Our findings also indicate that trauma-focused psychological therapies were effective treatments for PTSD following perinatal loss and perinatal trauma. This is in line with previous studies that found that EMDR and TF-CBT are effective treatments for perinatal trauma and loss by decreasing the frequency of flashbacks related to perinatal trauma or loss [46, 47]. Additionally, a reduction of flashbacks, likely facilitated by these therapeutic approaches, may have played a role in improving wellbeing and, in turn, alleviating overall psychological distress [46, 47].

The finding that psychologically informed midwifery support helps reduce distress and supports with positive birth outcomes is in line with literature showing that midwifery-led interventions, such as early identification of risk factors for birth trauma, can help empower individuals and prevent a traumatic birth [48]. Whilst the literature available is limited, some studies have suggested that specialist midwifery led care can improve the maternal experience and reduce birth trauma [49].

The results indicated that joint interventions of psychology and midwifery support helped reduce psychological distress, resulting in better experiences in clients’ maternity journey. This is consistent with previous studies that have shown that a trauma-informed, collaborative approach promotes an individual’s control and choice [50] and highlights the importance of including all professionals in a client’s care to ensure their mental, physical, emotional and social needs are met.

All feedback given to the MTLC service via the Service User Satisfaction Survey was positive. Quantitative results revealed all clients who answered the survey felt they were very involved in making decisions about their care and treatment whilst under the MTLC service and clients felt treated with kindness and respect by MTLC staff, suggesting that the service met two important principles of TIC. The qualitative findings from the survey suggest that clients felt supported, listened to and that MTLC midwives advocated for them in maternity spaces. This in turn increased clients’ confidence and sense of safety, a factor that has been shown to play a protective role in preventing traumatic birthing experiences [37]. Qualitative results showed that space was held for clients to acknowledge their perinatal loss and grief, which is often societally unrecognised, and experienced as disenfranchised grief [51]. Given perinatal loss is a common experience [21] and there is a lack of services offering specialist support for individuals effected by perinatal loss, MTLC is an example of an MMHS that fills this gap.

In addition, MTLC addresses cultural sensitivity and inclusivity. In a context where Black women are more than twice as likely to die during or after pregnancy as White women, the service makes cultural safety explicit: birth plans are co-produced, partners are included, and language, faith and migration histories are explored from assessment onward. NHS England also explicitly frames perinatal TIC as inclusive of all birthing people, which MTLC adopts.

MTLC offers culturally adapted TFCBT and EMDR with interpreters where needed, midwife–psychology joint work to advocate within maternity units, and staff training in anti-racism and reflective practice. MTLC stratify outcomes and access by ethnicity and borough. A quality improvement project is underway in MTLC to establish how many people from ethnic minority groups who experience a perinatal loss are offered and take up referral to MTLC; findings will inform targeted outreach, clearer referral prompts in maternity notes, and co-designed information materials.

### Strengths and limitations

Overall, the study found most clients who engaged with treatment in the MTLC service experienced a significant reduction in psychological distress, PTSD symptoms, low mood and anxiety and had positive experiences with the service. However, these results need to be interpreted with caution because of the following limitations: a limited number of qualitative responses meant that a structural qualitative analysis could not be conducted to identify themes. At the time of the research, no outcome measure was used to help quantify levels of grief experienced by clients, so MTLC were unable to draw inferential conclusions about changes to individual’s experience of grief following perinatal loss before and after treatment.

Only 17 women completed the Service User Satisfaction Survey, therefore positive results might not be reflective of the entirety of the population who received the service. In addition, a significant number of clients dropped out of treatment or disengaged with the service. Whilst they were sent the Service User Satisfaction Survey, no responses were received. A quality improvement project obtaining feedback on why clients disengaged may have been helpful to identify barriers to engagement. A recent progress report stated MMHS’ are underfunded and spread far too thinly to meet the level and intensity of demand [52]. This has been the experience for MTLC where the demand supersedes the current resource, resulting in the service needing to operate waiting lists.

Despite the limitations, this study had several strengths. The availability of our ethnicity data is a strength to the study, as our sample is ethnically diverse and therefore provides a better understanding of treatment for tokophobia, perinatal loss and perinatal trauma. Moreover, the novelty of this research is a strength, as, to our knowledge, this is the first of its kind to evaluate a service that offers specialist assessment and treatments for those affected by perinatal trauma, perinatal loss and Tokophobia. Quantitative analysis showed objective changes in PTSD symptoms and psychological distress before and after treatment, lending weight to psychological and midwifery support in Maternal Mental Health Services.

### Clinical implications

Our findings demonstrate that offering early access to trauma-focused psychological interventions within maternity care can significantly reduce symptoms of PTSD, anxiety, and psychological distress. This underlines the value of embedding psychological services within maternity pathways, especially for those at risk of perinatal trauma or loss. Additionally, the role of trauma-informed midwifery care and continuity of care appeared pivotal in this study and suggest that if introduced at a wider scale in maternity services could avoid re-traumatisation. Lastly, the results suggest that trauma-informed approaches are effective and accessible across different cultural groups. Given persistent disparities in maternal outcomes, especially among Black and Asian women, the clinical model used in MTLC may be key to reducing inequality in maternal mental health outcomes.

### Positioning services within broader maternal mental health care

Prior to the introduction of maternal mental health services under the NHS Long Term Plan, many women and birthing people with perinatal trauma, loss, or severe fear of childbirth fell outside the remit of existing services and so were unable to receive trauma-informed specialist care. Community perinatal mental health services focus primarily on severe and enduring mental illness and NHS Talking Therapies are designed for common mental health conditions.

In contrast, MTLC offers specialist care to manage complex trauma linked to the maternity journey. This is especially critical in ethnically diverse areas like West London, where high levels of healthcare inequalities intensify the need for a service model that allows clients to receive support that acknowledges factors such as cultural context, historical trauma, and systemic barriers, which are not routinely addressed elsewhere.

## Conclusions

This evaluation of a MMHS found that trauma-informed psychological therapy and midwifery support for women and birthing people experiencing perinatal trauma, loss or fear of childbirth, led to a significant reduction in psychological distress and PTSD symptoms. Clients who provided feedback felt well supported, advocated for in maternity settings and more confident, in line with the principles of culturally sensitive TIC. There needs to be further research on the potential value of continuity of midwifery care regarding reducing birth trauma and subsequent mental health morbidity in the postnatal period. Future research could also focus on developing evidence-based interventions for treatment of those experiencing perinatal loss as there is limited literature on this. In addition, considerations could be made around TIC training in maternity services in the hope of preventing the need of MMHS’. In this way, staff on the front line would be able to embed TIC into practice and care, in the hope that this would reduce emotional distress for those experiencing tokophobia, perinatal loss or perinatal trauma.

## Supplementary Information

Additional file 1.

## Data Availability

The datasets used and/or analysed during the current study are available from the corresponding author on reasonable request.

## Abbreviations

MTLC: Maternity Trauma and Loss Care Service
PTSD: Post-traumatic Stress Disorder
TIC: Trauma-informed care
PCL-5: Post-traumatic Stress Disorder Checklist for DSM-5
TF-CBT: Trauma-Focused Cognitive Behavioural Therapy
EMDR: Eye Movement Desensitisation and Reprocessing
