# Supplementary material for: Transforming care by integrating maternity and psychological support: a mixed-methods evaluation of a Maternal Mental Health Service
Source: BMC Pregnancy Childbirth. 2025 Oct 17;25:1111. doi: 10.1186/s12884-025-08096-9 (PMC12534978; doi:10.1186/s12884-025-08096-9)
Supplement: Supplementary file 1 — Additional file 1. [file 12884_2025_8096_MOESM1_ESM.docx]

**Supplementary Table** Ethnicity of clients offered an assessment (*n* = 185)

| Ethnicity |  |
| --- | --- |
| White - British | 46 |
| White - Other | 41 |
| Mixed - White and Black | 1 |
| Mixed - Other | 13 |
| Asian – Indian | 22 |
| Asian or Asian British - Other | 14 |
| Black or Black British | 13 |
| Black or Black British - Other | 3 |
| Other Ethnic Groups - Any other ethnic group | 17 |
| Not stated | 15 |
